# Cancer and Cancerous Diseases

**Published:** 1888-02

**Authors:** 


### *CANCER AND CANCEROUS DISEASES.*

In the Morton Lecture, recently delivered before the Royal College of Surgeons, of England, Sir James Paget pointed out a number of interesting and instructive analogies between the processes of plant and animal life, as calculated to throw light on the nature of cancer, and also some interesting speculations as to a future possible cure for it. Mr. Paget contends that the whole study of tumors may, indeed, find admirable illustration in vegetable pathology. The xylomas or tumors of homologous woody tissue he compares to the benign tumors found in human pathology, while the many varieties of galls may be taken to represent malignant growths. The xylomas are outgrowths from the healthy wood that seems to have received no injury, and are apparently developments from adventitious or latent buds, while the galls are morbid growths that result from the stings of various insects, just as benign growths in the human organism are developments of what may be considered latent embryonic tissue, and the cancerous and other essentially harmful local manifestations of disease may be considered as indicating the results of irritation by a specific virus. The galls are all the result of the stings of insects, and they amount, in all, to more than a thousand varieties. Each insect, with an instinct as unfailing as natural law, deposits its eggs, with the accompanying virus, on a leaf or other part of the very plant in which the right kind of gall can be formed. Each virus requires a susceptible and fitting place or substance. Something like this, Paget believes to be the case in cancerous diseases. Cancer requires first the system capable of the proper reaction, the condition of blood, etc., in which cancer can flourish, and then the specific virus that alone can produce cancer. In this position, the eminent teacher seems to be thoroughly established and fortified.

But, leaving the exact line of argument of Mr. Paget, it is worth while to ask how much of the method and sequence of reaction is due to the virus in all infectious diseases, and how much to the capabilities of the system for reaction ?

In the first place, a benign woody tumor is not the exact counterpart of a benign tumor in animal tissues. It is the function of many plants to form buds anywhere beneath the bark, and of some in any cells, even in the leaves. These adventitious buds might anywhere become aborted shoots or twigs; they are the counterparts of true moles among animals, or possibly of dermoid cysts. The illustration drawn from galls has a better application. In these instances, then, we might inquire what part is performed by the virus, and what part by the organism? Does the virus which the insect deposits as it lays its egg enter into the vital forces of the tree to determine a gall of a certain shape, size and chemical constitution, or does the tree or plant of itself possess the inherent power of reacting in a particular manner, which results in the production of a certain character of gall or other growth? Taken in the human body, does the virus of small-pox join in the definite processes that result in fixing the duration of the fever, the time of the outbreak, and all the rest of the orderly processes observed in a case of small-pox, or do these powers belong to the system, to be called forth in different ways, according to the nature of hurt received by it?

We think they belong to the organism, and not to the virus. The virus is almost passive. The case seems to be not unfitly represented by the siege of a fort. An enemy approaches to take it, and upon the movements of the enemy, upon the nature of the attack, will depend the character of the defense. Which cannon shall be fired, where a ditch shall be dug, where pickets shall be placed, where a mine shall be exploded—all depend upon the position of the enemy, but the people in the fort do all of these things of themselves. As many obvious means of defense as the human organism possesses, as many words, as many different possible movements, either of definite expression or otherwise—they probably do not all approximate in number and variety the vital reactions possessed by the cells of the body. Cancer, then, in our opinion, as it presents itself to sight, is an effort of the system to throw off a foreign hostile substance of which we know, so far, absolutely nothing. It may be a microbe, it may be degenerate bioplasm, or it may have some other nature, still, only this we know: it is hostile.

It would be unprofitable, perhaps, to carry out the long train of speculation to which this subject might lead. What we want most is a cure, a specific, as pointed out by the great lecturer, like that which mercury furnishes for syphilis, and quinine for malaria. And, whatever else is done, something in the way of medication should be tried for every cancer. If it cannot be operated on, something not already a proven failure should be tried for its cure; if it can be, or has been operated upon, then for the prevention of its return.—*American Practitioner and News.*

